# Lack of Pharmacokinetic Drug–Drug Interactions Between Bepirovirsen and Nucleos(t)ide Analogs

**DOI:** 10.1002/cpdd.1518

**Published:** 2025-02-14

**Authors:** Kelong Han, Amir S. Youssef, Mindy Magee, Steve Hood, Helen Tracey, Jesse Kwoh, Dickens Theodore, Melanie Paff, Ahmed Nader

**Affiliations:** ^1^ GSK, Clinical Pharmacology Modeling and Simulation Collegeville Pennsylvania USA; ^2^ GSK, DMPK ‐ Disposition & Biotransformation Stevenage Hertfordshire UK; ^3^ GSK, PBPK Modelling, DMPK, Preclinical Sciences, Research Technologies, R&D Stevenage Hertfordshire UK; ^4^ Ionis Pharmaceuticals Inc. Carlsbad California USA; ^5^ GSK, Clinical Research, Hepatology Durham North Carolina USA; ^6^ GSK, Development Medicine Collegeville Pennsylvania USA

**Keywords:** bepirovirsen, chronic hepatitis B, drug–drug interaction, nucleos(t)ide analogs, pharmacokinetics

## Abstract

Bepirovirsen is an antisense oligonucleotide currently in Phase 3 development to treat chronic hepatitis B virus (HBV) infection. Given the importance of coadministration of bepirovirsen and standard‐of‐care nucleos(t)ide analogs (NAs), we evaluated drug–drug interactions (DDIs) between bepirovirsen, entecavir (ETV), and tenofovir (TFV) using in vitro and clinical data obtained through innovative study design and sampling strategy. Static models employing in vitro data indicated that bepirovirsen is not a direct inhibitor or inducer of most drug‐metabolizing enzymes or an inhibitor or substrate of drug transporters and poses no clinical DDI risk against NAs. Bepirovirsen plasma pharmacokinetic parameters and concentration–time profiles in patients with chronic HBV in the CS3 study (NCT02981602) were similar with or without ETV or TFV coadministration, indicating no effect of NA coadministration on bepirovirsen pharmacokinetics. In patients with chronic HBV receiving both bepirovirsen and ETV or TFV in the B‐Clear study (NCT04449029), NA plasma concentrations and pharmacokinetic parameters were similar to those published without bepirovirsen coadministration, suggesting no effect of bepirovirsen coadministration on NA pharmacokinetics. This analysis demonstrated no DDI potential between bepirovirsen and NAs, suggesting that dedicated clinical DDI studies are not required. Bepirovirsen is currently being evaluated in Phase 3 studies in combination with NA.

Chronic hepatitis B virus (HBV) infection is a major worldwide health concern, with an estimated 258 million individuals living with chronic HBV infection in 2022.^1^ Despite the high prevalence of chronic HBV infection in many parts of the world, a modeling study conducted across 170 countries showed that only about 14% of individuals with chronic HBV infection are diagnosed globally (36.0 million), and among those eligible for treatment, only 8% receive treatment (6.8 million of the estimated 83.3 million eligible for treatment) in 2022.^1^ Furthermore, it is projected that the number of annual HBV‐related deaths will rise globally from 858,000 in 2015 to 1,149,000 in 2030.^1^

The most frequently prescribed standard‐of‐care therapies for patients with chronic HBV infection is nucleos(t)ide analogs (NAs).^2^ Entecavir (ETV) and tenofovir (TFV) are the most commonly used NAs^2^, ^3^; they inhibit HBV polymerase by directly competing with the natural deoxyribose substrate, thereby suppressing HBV replication.^4^, ^5^ Durable hepatitis B surface antigen (HBsAg) loss (<0.05 IU/mL) and undetectable serum HBV DNA at 24 weeks off treatment, known as functional cure, has become the recommended outcome for novel HBV therapies.^6^, ^7^, ^8^ However, HBsAg loss is rarely achieved with the current standard of care (HBsAg loss after 48 weeks of treatment: 1% or less for NA).^2^, ^9^

Although current therapies are unable to reliably achieve functional cure, several new compounds are under investigation for the treatment of chronic HBV infection.^10^ One such compound is bepirovirsen, an unconjugated antisense oligonucleotide (ASO) currently in Phase 3 development as a novel therapeutic agent.^11^, ^12^ Bepirovirsen binds to a complementary sequence present in all HBV messenger RNA transcripts and targets all HBV RNAs (including pregenomic RNA), impacting HBV infection in 3 ways: reductions in viral proteins, including HBsAg; reductions in HBV DNA; and stimulation of the immune system, potentially via Toll‐like receptor 8 activation.^12^, ^13^, ^14^, ^15^, ^16^ In the Phase 2b B‐Clear study, treatment with bepirovirsen 300 mg for 24 weeks induced HBsAg and HBV DNA loss, which was sustained for 24 weeks after the end of bepirovirsen treatment in 10% of participants who were not on NA therapy and in 9% of participants who were on stable NA at study entry and remained on NA therapy after bepirovirsen cessation.^12^

ASOs are anticipated to have low drug–drug interaction (DDI) potential based on their metabolic pathways.^17^ This has been empirically confirmed by the lack of clinically meaningful DDIs in several published studies of ASOs, both in vitro and in vivo.^18^, ^19^, ^20^, ^21^, ^22^, ^23^ These studies demonstrated a lack of interaction between ASOs and a number of therapies, including antidiabetic compounds (eg, metformin, glipizide, or rosiglitazone),^18^ warfarin,^19^ combination cisplatin and gemcitabine,^20^ simvastatin and ezetimibe,^21^ and major drug transporters.^22^ Furthermore, ASOs are not cytochrome P450 (CYP) substrates and because of their low affinity toward CYP enzymes, no DDIs with other small molecules have been reported for any clinical ASO drug thus far.^24^ Therefore, it is likely that bepirovirsen has a low DDI risk and is not expected to have an impact on the pharmacokinetics (PK) of NAs. However, given the importance of coadministration of bepirovirsen and NAs, the current study characterized the DDI potential between bepirovirsen and NAs using an innovative study design and sampling strategy to further substantiate the contention that evaluation of DDI potential may not require dedicated clinical DDI studies for bepirovirsen.^22^

## Methods

The DDI potential between bepirovirsen and NAs was assessed using both in vitro and clinical data:

- In vitro assessment of the potential of bepirovirsen as a clinically relevant DDI perpetrator and victim, that is, as inhibitor and/or inducer of drug‐metabolizing enzymes and as inhibitor and/or substrate of drug transporters.
- Clinical evaluation of bepirovirsen as a DDI victim (effect of NA on bepirovirsen PK) in the Phase 2 CS3 study (NCT02981602; GSK study 205695).
- Clinical evaluation of bepirovirsen as DDI perpetrator (effect of bepirovirsen on NA PK) in the Phase 2b B‐Clear study (NCT04449029; GSK study 209668).

### In Vitro Interaction Between Bepirovirsen, CYP, and Uridine Diphosphate–Glucuronosyltransferase Enzymes and Drug Transporters

The reversible and time‐dependent inhibition of CYP1A2, CYP2B6, CYP2C8, CYP2C9, CYP2C19, CYP2D6, and CYP3A4/5; the induction of CYP1A2, CYP2B6, and CYP3A4; and the inhibition of uridine diphosphate–glucuronosyltransferase (UGT) enzymes were investigated in cryopreserved human hepatocytes using sensitive probe substrates. Method details are reported in the Supplementary Methods Sections 1.1 and 1.2.

Bepirovirsen was evaluated as an inhibitor and/or substrate of the drug transporters organic anion‐transporting polypeptides (OATP)1B1 and OATP1B3, organic anion transporters (OAT)1 and OAT3, organic cation transporters (OCT)1 and OCT2, multidrug and toxin extrusion transporters (MATE)1 and MATE2‐K using human embryonic kidney mammalian (HEK293) cells expressing these transporters, as an inhibitor and/or substrate of P‐glycoprotein (P‐gp) and human breast cancer resistance protein (BCRP), and as an inhibitor of multidrug resistance–associated protein 2 (MRP2) using inside‐out membrane vesicles prepared from cells overexpressing human adenosine triphosphate–binding cassette (efflux) transporters. Method details are reported in the Supplementary Methods Section 1.3.

The in vitro assessment was further used to assess the potential effect of bepirovirsen as a modulator (inhibitor or inducer) of enzymes and transporters using basic static models^25^, ^26^, ^27^, ^28^, ^29^, ^30^ for quantitative predictions of DDIs, where applicable.

Model parameters including reversible inhibition (half‐maximal inhibitory unbound concentration [IC_50,u_]), or induction (unbound concentration causing half‐maximal induction effect [EC_50,u_] and maximum induction effect [E_max_]) of the mechanism in question (eg, CYP) and the contribution of clearance and fraction metabolized for selective substrates of drug‐metabolizing enzymes (CYPs) were applied where applicable are shown in Table S1. Equations and cutoff values were reported in the literature (Supplementary Methods, Sections 1.4 and 1.5).^25^, ^26^, ^27^, ^28^, ^29^, ^30^

As bepirovirsen is rapidly distributed into the liver,^31^ an analysis of the most relevant concentration to use in the static modeling was performed to inform the perpetrator of DDI risk for bepirovirsen. The maximum unbound plasma concentration (C_max_), following the therapeutic subcutaneous dose of 300 mg, administered weekly for 24 weeks, was 57 nM. Based on extrapolations from concentration measurements in preclinical species, the total liver concentration of bepirovirsen at steady state was estimated to be 34.8 µM, which seems, on first appearance, to be higher than the plasma. However, when the distribution between the hepatocytes and nonparenchymal cells is factored in, only 15% (5 µM) of the liver concentration is associated with the hepatocytes. Furthermore, multiple sources estimate that greater than 98% of the concentration of the drug in the hepatocytes is sequestered in the endo‐lysosomal system,^32^, ^33^, ^34^ resulting in an unbound concentration of 5 nM of bepirovirsen in the cytoplasm. The unbound concentration of bepirovirsen in the hepatocyte that is available for interaction with CYPs, UGTs, and transporters was estimated to be approximately 10‐fold lower than the unbound plasma C_max_ of 57 nM. The unbound plasma concentration was therefore used in the models to provide a conservative and robust assessment of DDI risk.

### Clinical Evaluation of Bepirovirsen as DDI Victim (Effect of NA on Bepirovirsen PK) in the CS3 Study

#### CS3 Study Design

CS3 was a Phase 2, double‐blinded, randomized, placebo‐controlled dose‐escalation study to examine the safety, tolerability, PK, and antiviral activity of bepirovirsen in participants with chronic HBV infection.^11^, ^15^ The study design has been previously published.^11^, ^15^ Briefly, the study included 3 cohorts of treatment‐naïve participants (Cohort 1‐3, n = 24) who received bepirovirsen or placebo without NA, and 1 cohort of participants already on stable NA regimens (On‐NA; Cohort 4, n = 7) who continued to receive NA while receiving bepirovirsen or placebo.^15^ Treatment‐naïve participants were randomized to placebo, bepirovirsen 150 mg (Cohort 1), or 300 mg (Cohorts 2 and 3), and On‐NA participants to placebo or bepirovirsen 300 mg (Cohort 4). Participants received 6 doses of bepirovirsen or placebo, administered via subcutaneous injection, during the 4‐week treatment period: twice weekly during Weeks 1 and 2 (Days 1, 4, 8, and 11) and once weekly during Weeks 3 and 4 (Days 15 and 22). Bepirovirsen PK samples were intensively collected before dosing and at 0.5, 1, 1.5, 2, 3, 4, 5, 6, 24, 72, and 168 hours after bepirovirsen injection on Days 1 and 22. PK samples at 168 hours after the first dose (Day 1) were not collected because the second dose was given on Day 4 (96 hours after first dose). The study design of CS3 allows for comparison of bepirovirsen PK with (On‐NA participants) and without coadministered NA (treatment‐naïve participants).

#### Noncompartmental Analysis of Bepirovirsen in the CS3 Study

Bepirovirsen plasma PK parameters were calculated on Day 1 and Day 22 using noncompartmental analysis (NCA) methods with Phoenix WinNonlin Version 8.0 (Pharsight Corporation). PK parameters included C_max_, time to maximum plasma concentration (t_max_), area under the plasma concentration–time curve (AUC) from time zero to 24 hour after dosing, and AUC from time zero to the end of the dosing interval. Calculations were based on the actual sampling times.

### Clinical Evaluation of Bepirovirsen as DDI Perpetrator (Effect of Bepirovirsen on NA PK) in the B‐Clear Study

#### B‐Clear Study Design

B‐Clear was a Phase 2b, multicenter, randomized, partial blind, parallel study assessing the efficacy and safety of bepirovirsen in 2 populations of participants with chronic HBV infection: those on stable NA therapy and those not currently receiving NAs.^12^ The design of the study has been previously published.^11^, ^12^ NA PK samples were intensively collected before dosing and at 0.5, 1, 1.5, 2, 3, 4, 5, 6, 8, 12, and 24 hours after bepirovirsen injection in On‐NA participants who received NA daily and bepirovirsen subcutaneously weekly. NA PK samples were collected at least 6 weeks after the first bepirovirsen injection, allowing sufficient time for any potential induction or inhibition. This design allowed for the comparison of NA PK with coadministered bepirovirsen (intensive PK in B‐Clear) against published NA PK without coadministered bepirovirsen and against simulated PK profiles using published population PK models for ETV and TFV (administered as tenofovir disoproxil fumarate [TDF]) without coadministered bepirovirsen.^35^, ^36^, ^37^

#### NCA of NA in the B‐Clear Study

NA plasma PK parameters were calculated using NCA methods with Phoenix WinNonlin Version 8.1.0.3530. PK parameters included C_max_, AUC from time zero to 24 hours after dosing, and plasma concentration at the end of the dosing interval. Calculations were based on the actual sampling times.

#### Simulations

Plasma concentrations of NA (ETV and TFV) without coadministered bepirovirsen were simulated using published population PK models of NA and compared with those observed in the B‐Clear study with coadministered bepirovirsen.

##### ETV Population PK Model

Simulations were performed on the basis of the population PK models of ETV from Zhu et al. and Chan et al.^35^, ^37^ The model from Zhu et al. was a 2‐compartment PK model with first‐order absorption and elimination parameterized in terms of apparent clearance (CL/F), the volume of distribution of the central compartment, intercompartmental clearance (Q/F), and volume of distribution of the peripheral compartment following extravascular administration.^37^ Dose was a covariate on Q/F, and creatinine clearance (CRCL) normalized to ideal body weight–corrected CRCL was a covariate on CL/F. The model by Chan et al. was a 2‐compartment model with age on absorption rate constant, weight (normalized to 70 kg) on CL/F, CRCL on CL/F, and dose on Q/F.^35^

##### TDF Population PK Model

One published population PK model of TDF is available in the literature. This model was developed in patients with HIV and published by Jullien et al.^36^ It is a 2‐compartment model with first‐order absorption and elimination parameterized in terms of CL/F, volume of distribution of the central compartment, Q/F, and volume of distribution of the peripheral compartment.^36^ Covariates on CL/F included body weight and serum creatinine. There is no published population PK model of TFV after tenofovir alafenamide (TAF) administration in the literature.

##### Visual Predictive Checks

For each NA model, simulations were conducted using the individual participant dosing and covariates information from the participants in the B‐Clear study (500 replicates). Any 24‐hour postdose NA plasma concentrations that were collected after the next NA dose were excluded. Simulations were conducted using NONMEM version 7.3 (ICON Development Solutions). Visual predictive checks (VPCs; with median, 5th, and 95th percentiles of the prediction interval) were produced from the simulated concentration–time data using R version 4.1.1 (R Foundation for Statistical Computing) and R packages VPC Version 1.2.2.9000 and tidyVPC Version 1.3.0. Observed NA concentration data from B‐Clear participants were overlaid on the population PK model‐based simulations (VPCs) and compared using visual inspection.

### Study Conduct and Ethics

The CS3 and B‐Clear studies were conducted in accordance with the Declaration of Helsinki and the Good Clinical Practice guidelines of the International Council for Harmonization, and all applicable laws and regulations in participating countries. The protocol and amendments were reviewed and approved by local institutional review boards or independent ethics committees. Written informed consent was obtained from all participants involved in both studies.

### Bioanalytical Methods

PK samples in the B‐Clear study were assayed for bepirovirsen, ETV, and TFV in plasma at the bioanalytical facility Labcorp Drug Development, Shanghai, China, using validated liquid–liquid extraction for bepirovirsen, solid‐phase extraction for ETV, and protein precipitation for TFV assays followed by high‐performance liquid chromatography with mass spectrometric detection. All plasma samples were assayed within the established long‐term frozen, freeze/thaw, and processed extract stability. A summary of the bioanalytical performance of each analyte is presented in Table S2.

Blood samples in the CS3 study were collected from each participant in all treatment groups to determine the concentrations of bepirovirsen, ETV, and TFV using validated liquid–liquid extraction, solid‐phase extraction, and protein precipitation, respectively, followed by analysis using high‐performance liquid chromatography with tandem mass spectrometric detection. A complete description of the bioanalytical assay is presented in Table S3.

## Results

### In Vitro Interaction Between Bepirovirsen, CYP, and UGT Enzymes and Drug Transporters

Bepirovirsen was not a clinically relevant reversible or time‐dependent inhibitor of CYP1A2, CYP2B6, CYP2C8, CYP2C9, CYP2C19, CYP2D6, or CYP3A4/5 or a reversible inhibitor of UGT enzymes. Bepirovirsen inhibited CYP2C8 in vitro with a half‐maximal inhibitory concentration of >100 µM. Based on the results of the basic static model, bepirovirsen is not a clinically relevant reversible inhibitor of CYP2C8.

Bepirovirsen did not induce CYP1A2 or CYP2B6 in all 3 cryopreserved primary human hepatocyte donors. Bepirovirsen was an inducer of CYP3A4 in vitro in 1 of 3 cryopreserved human hepatocyte donors only, with unbound concentration causing half‐maximal induction effect and maximum induction effect values of 13.6 µM and 2.28‐fold, respectively (Table 1). The results of the basic static modeling indicate that bepirovirsen is not considered to be a CYP3A4 inducer of clinical DDI concern.

**Table 1:** Summary of Bepirovirsen In Vitro Perpetrator Drug Interaction Data

Enzyme/transporter	Direct inhibition (IC_50,u_ [µM]) and TDI	Induction (EC_50,u_ [µM] and E_max_)
CYP1A2	No inhibition observed	No induction observed
CYP2B6	No inhibition observed	No induction observed
CYP2C8	>100 µM No TDI	ND
CYP2C9	No inhibition observed	ND
CYP2C19	No inhibition observed	ND
CYP2D6	No inhibition observed	ND
CYP3A4	No inhibition observed	No induction observed (Donors 1 and 3) EC_50,u_ = 13.6 µM E_max_ = 2.28‐fold (Donor 2)
UGT	No inhibition observed	ND
P‐gp	No inhibition observed	ND
BCRP	No inhibition observed	ND
MRP2	8.49 µM^a^	ND
OATP1B1	No inhibition observed	ND
OATP1B3	No inhibition observed	ND
OAT1	No inhibition observed	ND
OAT3	No inhibition observed	ND
OCT1	No inhibition observed	ND
OCT2	No inhibition observed	ND
MATE1	No inhibition observed	ND
MATE2‐K	No inhibition observed	ND

BCRP, human breast cancer resistance protein; CYP, cytochrome P450; EC_50,u_, unbound concentration causing half‐maximal effect; E_max_, maximum induction effect; IC_50_, half‐maximal inhibitory concentration; MATE, multidrug and toxin extrusion transporter; MRP2, multidrug resistance‐associated protein 2; ND, not determined; OAT, organic anion transporter; OCT, organic cation transporter; OATP, organic anion transporting polypeptide; P‐gp, P‐glycoprotein; TDI, time‐dependent inhibition (conducted for CYPs only, not UGTs); UGT, uridine diphospho‐glucuronosyltransferase.

a Unbound concentration causing 50% of inhibition.

Bepirovirsen was not an in vitro inhibitor of P‐gp, BCRP, OATP1B1, OATP1B3, OAT1, OAT3, OCT1, OCT2, MATE1, or MATE2‐K. Bepirovirsen inhibited the MRP2 transporter in vitro with an IC_50,u_ of 8.49 µM. Results of the basic static model predicted that bepirovirsen is not a clinically relevant inhibitor of MRP2.

Bepirovirsen was not an in vitro substrate of MRP2, OATP1B1, OATP1B3, OAT1, OAT3, OCT1, OCT2, MATE1, or MATE2‐K. Bepirovirsen exhibited low permeability across the monolayers of the cells expressing the efflux transporter P‐gp or BCRP for both directions. As a result, reliable and meaningful efflux ratios could not be determined for P‐gp or BCRP.

### Clinical Evaluation of Bepirovirsen as DDI Victim (Effect of NA on Bepirovirsen PK) in the CS3 Study

**Figure 1:**
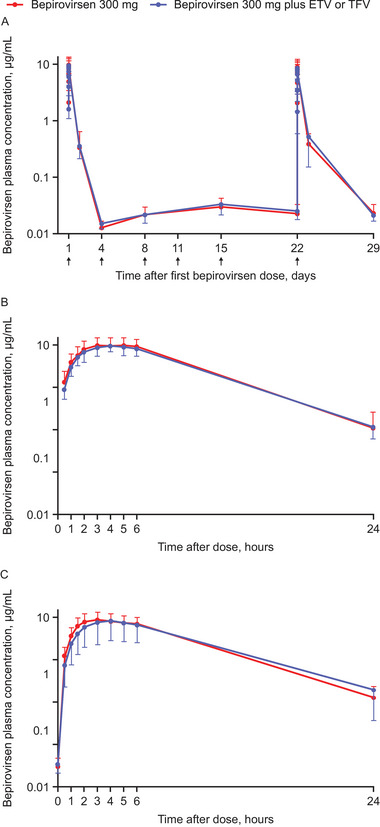
Mean plasma concentration of bepirovirsen with and without coadministered ETV or TFV (panel A shows all data; panels B and C show Day 1 and Day 22 data, respectively). Logarithmic scale. Error bars represent standard deviation. Arrows indicate bepirovirsen administration (Days 1, 4, 8, 11, 15, and 22). ETV, entecavir; TFV, tenofovir.

Bepirovirsen plasma concentration–time profiles were similar following subcutaneous administration of bepirovirsen 300 mg with or without coadministered ETV or TFV throughout the 4‐week treatment period (Figure 1A), both for the intensive sampling on Days 1 (Figure 1B) and 22 (Figure 1C) and the sparse sampling on other study days (Figure 1A).

Bepirovirsen plasma PK parameters for participants receiving bepirovirsen 300 mg with or without coadministered ETV or TFV, calculated on the basis of intensive sampling on Days 1 and 22, are summarized in Table 2.

**Table 2:** Summary of Plasma PK Parameters of Bepirovirsen With and Without NA in the CS3 Study

Cohort	Study day	AUC_0–24_ (µg•h/mL)	AUC_tau_ (µg•h/mL)	C_max_ (µg/mL)	t_max_ (hour)
Bepirovirsen 300 mg	Day 1 (n = 12)	134 (44.2) [61.5‐185]	—	10.6 (3.4) [5.2‐14.4]	4.0 (1.3) [2.0‐6.0]
	Day 22 (n = 12)	117 (34.9) [77.4‐198]	146 (34.7) [91.1‐226]	9.5 (3.1) [6.4‐17.3]	3.4 (0.9) [2.0‐5.1]
Bepirovirsen 300 mg plus ETV or TFV	Day 1 (n = 5)	123 (33.1) [80.1‐161]	—	9.7 (2.2) [7.1‐12.6]	4.1 (0.4) [3.9‐4.8]
	Day 22 (n = 4)	111 (54.3) [50.1‐178]	147 (38.0) [110‐200]	8.8 (4.7) [3.2‐14.3]	4.2 (1.2) [3.0‐5.8]

Values are displayed as mean (SD) [minimum–maximum].

AUC_0‐24_, area under the plasma concentration–time curve from time zero to 24 hours after dosing; AUC_tau_, area under the plasma concentration–time curve from time zero to the end of the dosing interval; C_max_, maximum concentration; ETV, entecavir; NA, nucleos(t)ide analogs; PK, pharmacokinetic; SD, standard deviation; t_max_, time to maximum plasma concentration; TFV, tenofovir.

### Clinical Evaluation of Bepirovirsen as DDI Perpetrator (Effect of Bepirovirsen on NA PK) in the B‐Clear Study

The analysis included 186 ETV plasma concentrations from 16 participants treated with bepirovirsen and ETV, 70 TFV plasma concentrations from 6 participants treated with bepirovirsen and TDF, and 70 TFV plasma concentrations from 5 participants treated with bepirovirsen and TAF. As summarized in Table 3, mean ETV and TFV C_max_ and AUC values from participants in the B‐Clear study who received coadministration of bepirovirsen and NA (ETV, TDF, or TAF) were consistent with published data without coadministered bepirovirsen.^38^, ^39^, ^40^, ^41^

**Table 3:** Plasma PK Parameters of ETV and TFV With and Without Bepirovirsen Coadministration

	AUC_0‐24_ (ng•h/mL)	C_max_ (ng/mL)	C_tau_ (ng/mL)
**ETV**			
B‐Clear study, 0.5 mg once daily	24.4 ± 6.8	6.5 ± 2.3	0.5 ± 0.1
Mean ± SD (n)	(12)	(16)	(12)
0.5 mg once daily^40^, ^41^	—	4.2	0.3
**TFV (administered as TDF)**			
B‐Clear study, 245 or 300 mg once daily	2754 ± 661	319 ± 95.9	57.0 ± 17.1
Mean ± SD (n)	(5)	(7)	(5)
300 mg single dose	2290 ± 690	300 ± 90.0	—
Mean ± SD^39^			
**TFV (administered as TAF)**			
B‐Clear study, 25 mg once daily	308 ± 105	19.4 ± 5.9	10.3 ± 3.5
Mean ± SD (n)	(4)	(5)	(4)
25 mg once daily	400 (35.2)	30.0 (24.6)	10.0 (39.6)
Mean (CV %)^a^			

AUC_0‐24_, area under the plasma concentration‐time curve from time zero to 24 hour after dosing; C_max_, maximum concentration; C_tau_, concentration at the end of the dosing interval; CV, coefficient of variation; ETV, entecavir; PK, pharmacokinetic; SD, standard deviation; TAF, tenofovir alafenamide; TDF, tenofovir disoproxil fumarate; TFV, tenofovir.

^a^ Unpublished data appearing in the product label for TAF.

**Figure 2:**
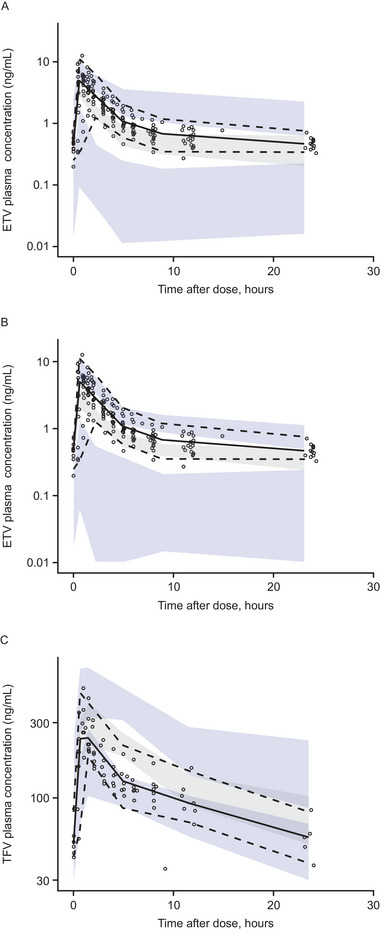
ETV (A and B) and TFV (C) plasma concentrations observed in B‐Clear with coadministered bepirovirsen and predicted by population PK models (A: Zhu et al.^37^; B: Chan et al.^35^; C: Jullien et al.^36^) without coadministered bepirovirsen. Semilogarithmic scale. Observed data: individual (dots), 5th (dashed), 50th (solid), and 95th (dashed) percentile. Predicted data (shaded): 5th (lower blue), 50th (gray), and 95th (upper blue) percentiles of the prediction intervals. ETV, entecavir; PK, pharmacokinetic; TFV, tenofovir.

VPC plots demonstrated a general agreement between observed ETV concentrations from participants in the B‐Clear study who received coadministration of bepirovirsen and predicted ETV concentrations without coadministered bepirovirsen from both the Zhu model^37^ (Figure 2A) and the Chan model^35^ (Figure 2B). The median of the observed data was slightly higher than the median prediction; however, more than 90% of the observed data fell within the 90% prediction intervals generated by the models.

The VPC plot demonstrated general agreement between the observed TFV concentrations in the B‐Clear study who received coadministration of bepirovirsen and TDF and predicted TFV concentrations without coadministered bepirovirsen from the Jullien model^36^ (Figure 2C). The median of the observed data was slightly lower than the median prediction; however, more than 90% of the observed data fell within the 90% prediction interval.

## Discussion

This analysis of bepirovirsen using in vitro evaluation of enzyme and transporter DDI data along with observed clinical PK data from participants with chronic HBV infection from the CS3 and B‐Clear clinical studies provides a valuable assessment of the DDI potential between bepirovirsen and NAs. This investigation demonstrated that bepirovirsen is not a clinically relevant inducer or inhibitor for a range of enzymes and transporters. Furthermore, the study highlighted that coadministration of ETV or TFV had no effect on bepirovirsen plasma concentrations, and coadministration of bepirovirsen with ETV, TDF, or TAF did not affect the systemic PK exposure of ETV or TFV. Thus, the analyses reported here showed no potential for DDI between bepirovirsen and ETV or TFV, substantiated by both in vitro assessments and clinical evaluation. Based on the totality of data, bepirovirsen Phase 3 studies were permitted by health authorities to proceed without conducting dedicated DDI studies.

ASOs are not typically metabolized by CYP enzymes but are instead primarily metabolized by endonucleases and exonucleases or have been chemically modified to resist degradation.^11^, ^17^, ^22^, ^24^, ^42^ Therefore, it is not expected that inhibitors or inducers of CYP enzymes will have an impact on their disposition. Furthermore, it is generally not expected that modulation of efflux transporters (eg, P‐gp, BCRP), hepatic uptake transporters (eg, OATP1B1, OATP1B3), or renal uptake or efflux transporters (eg, OAT1, OAT3, MATE1, MATE2/K) would have a significant impact on the PK of ASOs.^11^, ^17^, ^21^, ^24^, ^42^ For ASOs that undergo substantial renal active secretion as unchanged drugs, it could be important to evaluate whether they are substrates of renal transporters in vitro. As per data in the literature, which suggest a low likelihood of ASOs to be potent, direct inhibitors of drug‐metabolizing enzymes and transporters,^22^, ^43^ the in vitro findings showed that bepirovirsen at the therapeutic dose of 300 mg, with a corresponding estimated unbound C_max_ of 57 nM, was not a clinically relevant inducer of CYP3A4, reversible inhibitor of CYP2C8, or inhibitor of MRP2. Furthermore, all other enzymes and transporters investigated in vitro with bepirovirsen demonstrated no inhibition or induction. Although reliable and meaningful efflux ratios could not be determined for P‐gp or BCRP, bepirovirsen is unlikely to be a substrate of P‐gp or BCRP efflux transporters.

To test if NAs can impact bepirovirsen PK (bepirovirsen as victim), the Phase 2 CS3 study was used to compare bepirovirsen PK parameters and plasma concentration–time profiles in participants treated with bepirovirsen with and without coadministered ETV or TFV. The findings of this analysis showed that bepirovirsen plasma PK parameters and plasma concentration–time profiles were similar with and without coadministered ETV or TFV, demonstrating no effect of coadministered NAs on bepirovirsen plasma PK.

To evaluate whether bepirovirsen impacts the PK of NAs (bepirovirsen as perpetrator) observed NA (ETV and TFV) PK data from participants from the Phase 2b B‐Clear study who received coadministration of bepirovirsen and NA was compared against published PK data of NA administered without bepirovirsen. The results of this analysis demonstrated overall agreement of the NA PK with and without coadministered bepirovirsen, suggesting no effect of coadministered bepirovirsen on NA plasma PK.

The original purpose of the intensive PK sampling in B‐Clear was only to characterize bepirovirsen plasma PK; the sampling schedule was before dosing and at 1, 2, 3, 4, 5, 6, 8, 12, 24, 72, and 168 hours after dosing. However, valuable NA PK data were collected by making the following additions without adding an excessive amount of extra burden to the study participants. First, 2 plasma samples were added at 0.5 and 1.5 hours after dosing given that the t_max_ of ETV and TFV was approximately 1 hour after dosing. Second, the blood volumes of the samples collected within 24 hour after dosing were increased to allow measurement of NA plasma concentrations. Finally, all participants who consented to intensive PK collection were asked (and reminded) to take the NA tablet in the clinic after the predose sample was collected instead of taking the NA tablet at home, as they did every day. This was important given the short t_max_ of ETV and TFV, as well as the importance of capturing C_max_ during DDI assessment. If the participants had taken the NA tablet at home, as they usually did, NA C_max_ would have been missed by the time they arrived in the clinics on the day of intensive PK collection. This innovative study design and carefully planned execution enabled the collection of high‐quality NA PK data, which could potentially help in avoiding unnecessary DDI studies.

A key aspect of the current analysis is that it enabled DDI interactions to be assessed without the need for dedicated DDI studies. The CS3 study allowed for the comparison of bepirovirsen PK in treatment‐naïve participants and participants on stable NAs, and the B‐Clear study allowed for the comparison of NA PK from participants in the On‐NA cohort treated with bepirovirsen against published NA PK without bepirovirsen. Recently, a structural population PK model for bepirovirsen was developed on the basis of data from 3 previous clinical studies, including the CS3 and B‐Clear studies, which indicated that chronic HBV infection status and body weight were significant variables in the final model, whereas demographic or baseline characteristics, as well as concomitant NA treatment status, were not identified as significant covariates, further supporting the findings that bepirovirsen PK is not affected by NA coadministration.^44^

The findings from the current analyses contribute to the existing body of literature noting that ASOs generally have a very low risk of DDI.^23^, ^45^, ^46^ There is an increasing number of reports showing no notable clinical DDI with ASOs, such as mipomersen, inotersen, and others, including the current data on bepirovirsen.^18^, ^19^, ^21^, ^22^, ^46^ In general, the low clinical DDI potential for ASOs can be attributed to the low likelihood of interaction with drug‐metabolizing enzymes/transporters and the fact that ASOs primarily bind to hydrophilic sites on serum albumin.^23^, ^45^, ^46^, ^47^ This is in contrast to most other drugs, such as small molecules, that bind to hydrophobic sites, decreasing the likelihood of plasma protein binding–based DDIs between ASOs and hydrophobic drugs.^33^, ^45^, ^47^ Furthermore, bepirovirsen's binding affinity to plasma proteins is low and nonspecific, in line with reports on other ASO PKs, further substantiating the low clinical DDI potential of this class of compounds.^33^, ^45^, ^47^ Similarly, binding of NAs to plasma proteins is relatively weak, suggesting that plasma protein binding has little to no impact on DDIs with NAs.^48^ In addition, in vitro DDI studies, including the current study, indicate that bepirovirsen is not an inhibitor of human transporters.^22^ As ETV and TFV are primarily renally excreted via transporters,^39^, ^40^ and an impact of bepirovirsen on renal transporters is not expected, as indicated by the findings of this study, clinical DDIs between bepirovirsen and ETV or TFV are unlikely. Finally, changes in cytokine levels may affect DDI risk due to altered levels of CYPs and drug transporter.^49^ Considering that the concentration of bepirovirsen in the hepatocyte available for interaction with CYPs and transporters was estimated to be approximately 10‐fold lower than the unbound plasma C_max_ of 57 nM, following the therapeutic dose of 300 mg, the likelihood of cytokine induction in response to bepirovirsen is expected to be low.

There were several limitations of this analysis. First, the perpetrator (NA) and victim (bepirovirsen) were often not administered at the same time in the CS3 study because the participants took the NA tablet at home. However, considering that the NA tablet was taken daily, while bepirovirsen was injected weekly, the dosing time of NA as the perpetrator was unlikely to have a relevant impact on the analysis. Second, only the most common NAs used to treat chronic HBV infection (ETV and TFV)^3^ were evaluated as perpetrator and victim in this analysis. Other NAs were not used in the CS3 study, and NAs such as lamivudine and adefovir were used in only 4 of 227 (1.8%) On‐NA participants in B‐Clear.^12^ However, since other NAs often share the same elimination pathway as ETV and TFV (ie, predominantly renally eliminated),^50^ the conclusions from this analysis are anticipated to be applicable to other NAs. Third, the small sample size did not allow for any statistical comparison. Fourth, due to the lack of fully described models of TFV after TAF administration in the existing literature, VPCs were not constructed for TAF. However, the NCA PK parameters from 5 subjects receiving TAF in the B‐Clear study were similar to the published data on TAF (Table 3). Finally, this analysis is focused on PK interactions, and potential pharmacodynamic interactions between bepirovirsen and NAs for the treatment of chronic HBV infection may be explored in Phase 3 studies and future analyses.

## Conclusion

These analyses showed no potential for DDI between bepirovirsen and NAs, with no effect of bepirovirsen coadministration on systemic PK exposure of ETV or TFV, and no effect of coadministered ETV or TFV on bepirovirsen plasma concentrations, suggesting that dedicated clinical DDI studies between bepirovirsen and NAs are not required. The combination of in vitro methodology, population PK modeling and simulations, and innovative design of clinical studies enabled the characterization of the PK‐based DDI potential between bepirovirsen and NAs supporting the continued coadministration of the 2 in clinical studies in patients with chronic HBV infection.

## Conflicts of Interest

Kelong Han, Amir S. Youssef, Mindy Magee, Steve Hood, Helen Tracey, Dickens Theodore, Melanie Paff, and Ahmed Nader are employed by GSK and hold financial equities in GSK. Jesse Kwoh is employed by Ionis Pharmaceuticals Inc. and holds financial equities in Ionis.

## Funding

Study NCT02981602, currently sponsored by GSK (Study ID: 209668/NCT04449029), was at the time of the trial supported by GSK and sponsored by Ionis Pharmaceuticals, Inc. (ISIS 505358‐CS3). B‐Clear (Study ID: 205695/NCT02981602) was funded by GSK.

## Supporting information



Supporting Information

## Data Availability

Please refer to GSK weblink to access GSK's data‐sharing policies and as applicable seek anonymized subject‐level data via the link https://www.gsk‐studyregister.com/en/.
