# Supplementary material for: Lack of Pharmacokinetic Drug–Drug Interactions Between Bepirovirsen and Nucleos(t)ide Analogs
Source: Clin Pharmacol Drug Dev. 2025 Feb 14;14(4):281–91. doi: 10.1002/cpdd.1518 (PMC11975176; doi:10.1002/cpdd.1518)
Supplement: Supplementary file 1 — Supporting Information [file CPDD-14-281-s001.pdf]

Lack of pharmacokinetic drug-drug interactions between bepirovirsen and nucleos(t)ide analogs

Kelong Han[1]*, Amir S Youssef[1]*, Mindy Magee[1], Steve Hood[2], Helen Tracey[3], Jesse Kwoh[4], Dickens Theodore[5], Melanie Paff[1], Ahmed Nader[1†]

[1]GSK, Clinical Pharmacology Modeling and Simulation, Collegeville, Pennsylvania, USA; [2]GSK, DMPK - Disposition & Biotransformation, Stevenage, Hertfordshire, UK; [3]GSK, PBPK Modelling, DMPK, Preclinical Sciences, Research Technologies, R&D, Stevenage, Hertfordshire, UK; [4]Ionis Pharmaceuticals Inc., Carlsbad, California, USA; [5]GSK, Clinical Research, Hepatology, Durham, North Carolina, USA

*Contributed equally

†Fellow of the American College of Clinical Pharmacology

Corresponding author: Kelong Han, PhD

Address: 1250 S Collegeville Rd, Collegeville, PA 19426, USA

Email: kelong.x.han@gsk.com

Supplementary Materials

Supplementary Methods

1.1 Determination of cytochrome P450 (CYP) and uridine diphosphate (UDP)-glucuronosyltransferase (UGT) inhibition in suspended cryopreserved human hepatocytes

To evaluate the direct inhibition of CYP and UGT enzymes, bepirovirsen was incubated in duplicate with appropriate probe substrate in suspended cryopreserved human hepatocytes at 1 million cells/mL. Bepirovirsen and hepatocytes suspended in Williams' E+ medium were added to 48-well plates and equilibrated for 5 minutes at ambient temperature prior to the addition of the probe substrate. Incubations were conducted at approximately 37°C in a mixture of 95% air and 5% carbon dioxide (CO_2) with 95% humidity. Reactions were terminated after approximately 10 minutes by the addition of acetonitrile containing an internal standard. The samples were centrifuged at 920 × g for 10 minutes at 10°C, and the supernatant fractions were analyzed by liquid chromatography tandem mass spectrometry (LC-MS/MS). Positive control incubations, replacing bepirovirsen with a known direct inhibitor of each CYP enzyme or with a broad-spectrum inhibitor of UGT enzymes, were included.

To assess the time-dependent inhibition of CYP enzymes, bepirovirsen was preincubated in duplicate with hepatocytes in Williams' E+ medium at approximately 37°C for approximately 30 minutes. Incubations were initiated by adding an appropriate probe substrate and continued as described above. Incubations without bepirovirsen and those containing bepirovirsen but not preincubated, served as negative controls. Additionally, positive controls incubations, in which bepirovirsen was replaced with a known time-dependent inhibitor of each CYP enzyme, were included.

Supernatants were analyzed by LC-MS/MS for concentrations of probe substrate metabolite as described in section 1.1.1.

Bepirovirsen concentration (µM)	Enzyme	Probe substrate	Probe substrate (µM)*
0, 0.1, 0.3, 1, 3, 10, 30, and 100	CYP1A2	Phenacetin	90
	CYP2B6	Bupropion	90
	CYP2C8	Amodiaquine	7
	CYP2C9	Diclofenac	2
	CYP2C19	*S*-Mephenytoin	60
	CYP2D6	Dextromethorphan	10
	CYP3A4/5	Midazolam	3
	CYP3A4/5	Testosterone	60
	UGT†	4-Methylumbelliferone	70

*Concentrations based on K_m or S_{50} data determined previously in incubations with human liver microsomes.
†UGT inhibition was measured using a probe substrate that is metabolized by multiple UGT isoforms.
K_m, substrate concentration supporting half the maximum rate of an enzyme-catalyzed reaction; S_{50}, substrate concentration for allosteric kinetics which supports half the maximum rate of an enzyme-catalyzed reaction

1.1.1 Determination of metabolite concentrations by LC-MS/MS analysis

Samples were analyzed by multiple reaction monitoring LC-MS/MS methods using an appropriate SCIEX (Framingham, MA, USA) mass spectrometer equipped with a Shimadzu Nexera (Shimadzu, Kyoto, Japan) or Shimadzu Prominence LC system interfaced by electrospray ionization. Methods were validated for absolute quantitation of probe substrate metabolite, using authentic metabolite reference standards and deuterated metabolites as internal standards, as detailed below.

Enzyme	Metabolite monitored	Internal standard	Mode
CYP1A2	Acetaminophen	Acetaminophen-d4	Positive
CYP2B6	Hydroxybupropion	Hydroxybupropion-d6	
CYP2C8	*N*-Desethyl Amodiaquine	*N*-Desethyl Amodiaquine-d5	
CYP2C9	4′-Hydroxydiclofenac	4′-Hydroxydiclofenac-d4	
CYP2C19	4′-Hydroxymephenytoin	4′-Hydroxymephenytoin-d3	Negative
CYP2D6	Dextrorphan	Dextrorphan-d3	Positive
CYP3A4/5	1′-Hydroxymidazolam	1′-Hydroxymidazolam-d4	
CYP3A4/5	6β-Hydroxytestosterone	6β-Hydroxytestosterone-d3	
UGT	4-Methylumbelliferone glucuronide	Hydroxycoumarin glucuronide-d5	

Metabolites were quantified by back calculation of concentrations against duplicate calibration curves using the simplest appropriate weighting and regression algorithm based on analyte/internal standard peak area ratios. Half maximal inhibitory concentration (IC_{50}) values were calculated using the following equation:

Assessment	Equation
IC_{50} determinations	$$\mathrm{Y} = \mathrm{Min} + \frac{(\mathrm{Max} - \mathrm{Min})}{\left(1 + \left(\frac{\mathrm{Conc}}{\mathrm{IC}_{50}}\right)^{\mathrm{slope}}\right)}$$

Average data (i.e., percent of control activity) obtained from duplicate samples for each bepirovirsen concentration were used to calculate IC_{50} values.
Conc, concentration; IC_{50}, half maximal inhibitory concentration; Max, maximum; Min, minimum.

1.2 Determination of CYP induction in cryopreserved primary human hepatocytes

To evaluate the induction of CYP1A2, CYP2B6, and CYP3A4, bepirovirsen was incubated with plateable human hepatocytes (BIOIVT, Baltimore, MD, USA) from 3 individual donors. Cells were thawed and plated as per manufacturer's instructions. Final hepatocyte density was 1.4 x 106 cell/mL. After 24 hours at approximately 37°C in 5% CO_2, cells were dosed in triplicate with bepirovirsen (0.01–200 µM) or the prototypical inducers rifampicin (0.01–30 µM), phenobarbital (1500 µM), and omeprazole (100 or 150 µM), or the negative control inducer flumazenil (50 µM) and incubated at approximately 37°C for 48 hours. Media was removed and replaced with fresh dosing medium 24 hours after the initial dose. After the addition of fresh dosing solution to the triplicate wells containing bepirovirsen, an aliquot of media was collected at 0, 4, and 24 hours and quantified for bepirovirsen concentration by LC-MS/MS, with actual measured concentrations used to calculate kinetic parameters.[1] At the end of the 48-hour incubation period, media was removed from all wells and an assessment of the effect of bepirovirsen or positive control on messenger RNA (mRNA) was evaluated by measuring mRNA levels of each CYP gene (CYP1A2, 2B6 and 3A4) via TaqMan real-time polymerase chain reaction analysis.

Maximum fold induction (E_{max}) and the unbound concentration eliciting half-maximal induction ($EC_{50,u}$) were determined for bepirovirsen using the following nonlinear regression model:[1]

Sigmoidal 3-parameter nonlinear regression model	$$y = bottom + \frac{E_{max} - bottom}{1 + e\left[\frac{\left(-[I] - EC_{50,u}\right)}{h}\right]}$$

Where:

bottom is the lowest fold induction and is constrained to “1.0” to normalize to vehicle control
y is relative fold induction
h is the Hill slope
[I] is the test article (bepirovirsen) concentration
$EC_{50,u}$ is the unbound concentration causing half-maximal effect
E_{max} is the maximum fold induction

1.3 Assessment of bepirovirsen as an inhibitor or substrate of human drug transporters

1.3.1 Assessment of bepirovirsen as an inhibitor of MATE1, MATE2-K, OAT1, OAT3, OATP1B1, OATP1B3, OCT1, and OCT2

The inhibitory potency of bepirovirsen against solute carrier (SLC) drug transporters was evaluated in human embryonic kidney mammalian (HEK293) cells overexpressing the respective transporter drug transporters organic anion-transporting polypeptide (OATP)1B1, OATP1B3, organic anion transporter (OAT)1, OAT3, organic cation transporter (OCT)1, OCT2, multidrug and toxin extrusion transporters (MATE)1 and MATE2-K. Cells were cultured at approximately 37°C in a humidified atmosphere containing 5% CO_2 and were plated onto standard 96-well tissue culture plates at an appropriate cell density.

Before the experiment, the culture medium was removed, and the cells were washed twice with 100 µL of pre-warmed (approximately 37°C) assay buffer (Krebs-Henseleit [pH 8.0] for MATE1, MATE2-K; Hank's balanced salt solution [pH 7.4] for OAT3, OATP1B1, OATP1B3, OCT2, OAT1, and OCT1). Thereafter, cells were preincubated at approximately 37°C for 30 minutes in assay buffer containing bepirovirsen at concentrations of 0.046–100 µM for MATE1, MATE2-K, OAT3, OATP1B1, OATP1B3, and OCT2, and 0.023–50 µM for OAT1 and OCT1, appropriate solvents for solvent controls and a known inhibitor for each transporter. In wells containing MATE1 and MATE2-K, buffer was supplemented with 20 mM NH4Cl during the 30 minutes preincubation. After the preincubation step, solutions were removed. Uptake experiments were performed at approximately 37°C in 50 µL of assay buffer containing the appropriate probe substrate (metformin [10 µM] for MATE1, MATE2-K, OCT2; tenofovir [5 µM] for OAT1; E3S [estrone-3-sulfate; 1 µM] for OAT3; $E_2$17βG [estradiol-17-β-

glucuronide; 1 µM] for OATP1B1; CCK8 [1 µM] for OATP1B3; sumatriptan [1 µM] for OCT1) and bepirovirsen or solvent or control inhibitor (incubation times: 1 minute for MATE2-K, OCT2 and OAT3; 2 minutes for OAT1 and OATP1B3; 3 mins for MATE1, OATP1B1, OCT1).

After the experiment, cells were washed twice with 100 µL of cold appropriate buffer and lysed with 50 µL of 0.1 M NaOH. Radiolabeled probe substrate transport was determined by measuring an aliquot (35 µL) from each well by liquid scintillation counting.

1.3.2 Assessment of bepirovirsen as a substrate of MATE1, MATE2-K, OAT1, OAT3, OATP1B1, OATP1B3, OCT1, and OCT2

For substrate assessment of SLC drug transporters by bepirovirsen, HEK293 cells overexpressing the respective transporter were incubated for 2 and 20 minutes in the appropriate assay buffer with bepirovirsen (0.1, 1, and 10 µM) in the presence and absence of transporter-specific inhibitor to determine whether bepirovirsen was actively taken up into the cells.

Cells were cultured at approximately 37°C in a humidified atmosphere containing 5% CO_2 and were plated onto standard 96-well tissue culture plates at an appropriate cell density.

Before the experiment, the medium was removed, and the cells were washed twice with 100 µL of the appropriate assay buffer. Cellular uptake of bepirovirsen into the cells was measured by adding 50 µL of the appropriate buffer containing bepirovirsen and incubating them at approximately 37°C. Recovery samples were collected from the buffer containing bepirovirsen at the end of the incubation. Reactions were quenched by removing the buffer containing bepirovirsen and the cells were washed twice with 100 µL of cold buffer. Cells were lysed by adding 150 µL of methanol:buffer mixture (2:1) and incubating for 20 minutes at 4 ± 1°C. The amount of bepirovirsen in the cell lysate was determined by LC-MS/MS. The amount of protein in each well was quantified using the bicinchoninic acid assay kit for protein determination (Sigma-Aldrich, St. Louis, MO, USA).

A positive control incubation was performed for each transporter using the appropriate probe substrate as described in section 1.3.1.

1.3.3 Assessment of bepirovirsen as an inhibitor of BCRP, MRP2, and P-gp

For the assessment of inhibition of human breast cancer resistance protein (BCRP), multidrug resistance-associated protein 2 (MRP2) and P-glycoprotein (P-gp), bepirovirsen was incubated with membrane vesicle preparations (total protein: 50 µg/well for MRP2 and P-gp; 12.5 µg/well for BCRP) and the probe substrate (E3S [1 µM] for BCRP; $E_2 17\beta G$ [100 µM] for MRP2; NMQ [N-methyl quinidine; 10 µM] for P-gp). Incubations were conducted in the presence of 4 mM adenosine triphosphate (ATP) or adenosine monophosphate (AMP) to distinguish between transporter-mediated uptake and passive diffusion into the vesicles. Bepirovirsen dissolved in 0.75 µL of solvent, was added across a concentration range of 0.046 to 100 µM into 50 µL of the reaction mixture. Then the reaction mixtures were preincubated for 15 minutes at 32°C for BCRP and P-gp and at approximately 37°C for MRP2. Reactions were initiated by the addition of 25 µL of pre-warmed 12 mM MgATP (or 12 mM AMP in assay buffer as a background control). Reactions were quenched by the addition of 200 µL of ice-cold washing buffer followed by immediate filtration via glass fiber filters mounted on a 96-well plate (filter plate). The filters were washed (5 × 200 µL of ice-cold washing buffer), dried and the amount of substrate inside the filtered vesicles was determined by liquid scintillation counting. Incubations were performed replacing bepirovirsen with known inhibitors of BCRP, MRP2, and P-gp.

1.3.4 Assessment of bepirovirsen as a substrate of BCRP, MRP2, and P-gp

The uptake of bepirovirsen into membrane vesicles was determined using inside-out membrane vesicles (total protein: 50 µg/well) prepared from cells overexpressing human ATP-Binding Cassette (ABC) transporters as well as from control cells. Vesicles were incubated for 10 minutes in the respective assay buffer containing bepirovirsen at two concentrations (1 and 10 µM) with or without ATP or AMP, and with or without a known inhibitor of the respective transporter to determine whether bepirovirsen is actively transported into the vesicles. Reactions were quenched by the

addition of 200 µL of ice-cold washing buffer and immediate filtration via glass fiber filters mounted to a 96-well plate (filter plate). The filters were washed with 5 × 200 µL of ice-cold washing buffer. The amount of accumulated bepirovirsen retained inside the vesicles was determined by LC-MS/MS after washing the vesicles 2 times with 100 µL methanol:water (2:1).

1.3.5 Determination of bepirovirsen as a substrate of drug transporters by LC-MS/MS analysis

Samples containing bepirovirsen and the analytical standard were analyzed by high-performance liquid chromatography (HPLC) MS/MS with methodology described below:

- HPLC was performed by using a Thermo Scientific Vanquish Flex UHPLC. Chromatographic separation was achieved using a gradient of 0.1% triethylamine (TEA) and 2% hexafluoroisopropanol (HFIP) in HPLC water and 0.1% TEA and 2% HFIP in Acetonitrile on an Agilent Poroshell 120 3.0x50 mm 2.7 µm column. Samples were analyzed by a Thermo Scientific Q-Exactive Focus Mass Spectrometer.
- HPLC was also conducted by using an Agilent 1260 HPLC. Chromatographic separation was achieved using a gradient of 0.1% TEA and 2% HFIP in HPLC water and 0.1% TEA and 2% HFIP in Acetonitrile on a Poroshell 120 EC-C18 50 × 3.0 mm, 2.7 µm column. Samples were analyzed by an Agilent 6460A Triple Quadrupole Mass Spectrometer.

Raw data was integrated using Analyst software (Sciex) to calculate peak area ratios to construct the calibration curves from which the concentration of bepirovirsen in the study samples was determined. The low concentration samples (e.g., 100 nM dosing samples for the substrate assays) were concentrated to ensure quantification of the compound in the samples.

In vesicular transport inhibition and SLC transporter inhibition assays, the IC_{50} (µM) was calculated, where applicable. IC_{50} was defined as the concentration of test article (TA) required to inhibit maximal activity by 50%. IC_{50} values were derived from a four-parametric logistic equation (log(inhibitor) versus response – variable slope); the curve was fitted to the relative activity versus TA concentration plot using non-linear regression. Top (maximal response) and bottom (maximally

inhibited response) values were not constrained to constant values of 100 and 0, respectively, unless it is noted otherwise.

Assessment	Equation
IC_{50} determinations	$y = bottom + \frac{top - bottom}{(1 + 10^{((logIC_{50} - X) \times Hillslope)})}$

Where:

bottom is the maximally inhibited response
top is the maximal response
y is the response (decreases as X increases)
X is the log of concentration (TA media concentrations)
IC_{50} is the half-maximal inhibition

1.4 Static model equations and cut-off values for the potential of bepirovirsen to modulate CYP enzymes

Mechanism	Equation
Reversible inhibition of hepatic CYPs[2]	$1 + \left(\frac{I_{max,u}}{K_{i,u}}\right) \geq 1.02$
Induction of CYPs[3]	$R_3 = \frac{1}{\left[1 + d \times \left(\frac{(E_{max} \times 10 \times I_{max,u})}{(EC_{50,u} + 10 \times I_{max,u})}\right)\right]} \leq 0.8$

Where:

$I_{max,u}$ is the maximal unbound plasma concentration of bepirovirsen.

E_{max} is the maximum induction effect determined in vitro.

$EC_{50,u}$ is the unbound concentration causing half-maximal effect determined in vitro.

d is the scaling factor and is assumed to be 1.

$K_{i,u}$ is the unbound inhibition constant determined in vitro.

Model parameters are shown in **Supplementary Table 1**.

1.5 Static model equations and cut-off values for the potential of bepirovirsen to modulate transporters

Mechanism	Equation
Hepatic efflux (after SC or IV administration) and renal uptake and efflux[4-6]	$\frac{Imax_u}{IC_{50}} \geq 0.1$

IV, intravenous; SC, subcutaneous.

Where:

IC_{50} is the half-maximal inhibitory concentration.

$Imax_u$ is the maximal unbound plasma concentration of bepirovirsen.

Supplementary Results

Supplementary Table 1. Mathematical parameters used in the static model to evaluate bepirovirsen drug-drug interaction risk prediction

Parameter	Value	Definition
Molecular weight	7344	Molecular weight of bepirovirsen (free acid)
Dose (mg)<br>SC route of administration	300	Current therapeutic dose of bepirovirsen
Estimated human unbound C_{max} (nM)	57	Systemic unbound C_{max} (plasma)
CYP1A2	No inhibition observed	Inhibition effect
CYP2C8 IC_{50}* (μM)	>100	Inhibition constant
CYP2C9	No inhibition observed	Inhibition effect
CYP2C19	No inhibition observed	Inhibition effect
CYP2D6	No inhibition observed	Inhibition effect
CYP3A4	No inhibition observed	Inhibition effect
UGT	No inhibition observed	Inhibition effect
CYP1A2	No induction observed	Induction effect
CYP2B6	No induction observed	Induction effect
CYP3A4 E_{max}	2.28	Maximum induction effect (Donor 2 only)
CYP3A4 $EC_{50,u}$ (μM)	13.6	Unbound concentration causing half-maximal induction effect<br>(Donor 2 only)<br>Bepirovirsen did not induce CYP3A4 in Donors 1 & 3
P-gp	No inhibition observed	Inhibition effect
BCRP	No inhibition observed	Inhibition effect
OATP1B1	No inhibition observed	Inhibition effect
OATP1B3	No inhibition observed	Inhibition effect
MRP2 $IC_{50,u}$ (μM)	8.49	Unbound concentration causing half maximal inhibitory effect
MATE1	No inhibition observed	Inhibition effect

Parameter	Value	Definition
MATE2-K	No inhibition observed	Inhibition effect
OAT1	No inhibition observed	Inhibition effect
OAT3	No inhibition observed	Inhibition effect
OCT1	No inhibition observed	Inhibition effect
OCT2	No inhibition observed	Inhibition effect

*IC_{50}/2 was used as an estimate of Ki for CYP2C8 inhibition prediction. No induction observed (<2-old max response).

BCRP, human breast cancer resistance protein; C_{max}, maximum concentration; CYP, cytochrome P450; E_{max}, maximum induction effect; IC_{50}, half-maximal inhibitory concentration; MATE, multidrug and toxin extrusion transporter; MRP2, multidrug resistance-associated protein 2; OAT, organic anion transporter; OCT, organic cation transporter; OATP, organic anion transporting polypeptide; P-gp, P-glycoprotein; SC, subcutaneous; UGT, uridine diphospho-glucuronosyltransferase.

Supplementary Table 2. Summary of bioanalytical assay performance in the B-Clear study

Analyte	Matrix	Validated Assay Range (LLOQ to ULOQ)	Inter-Assay Precision (% CV)	Accuracy (% Bias)
Bepirovirsen	Plasma	1.00 to 1000 ng/mL	5.0 to 7.0	−3.0 to 4.0
ETV	Plasma	0.0400 to 16.0 ng/mL	1.8 to 10.1	−2.1 to 4.4
TFV	Plasma	1.00 to 500 ng/mL	3.6 to 9.4	−1.6 to 3.0

CV, coefficient of variation; ETV, entecavir; LLOQ, lower limit of quantification; TFV, tenofovir; ULOQ, upper limit of quantification.

Supplementary Table 3. Summary of bioanalytical assay performance in the CS3 study

	**Bepirovirsen**	**ETV**	**TFV**
Analytical matrix	K_2EDTA Plasma	K_2EDTA Plasma	K_2EDTA Plasma
Internal standards	ISIS440762	Entecavir -$^{13}C_2$,^{15}N	Tenofovir –d7
Validated range (LLOQ to ULOQ)	1.00 to 1000 ng/mL	0.04 to 16.0 ng/mL	1.00 to 500 ng/mL
Quality control levels (ng/mL)	3.0, 30.0, 480, 800	0.12, 0.96, 6.4, 12.8	3.0, 30.0, 250, 400
Analytical technique/ method of detection	Liquid-liquid extraction	Solid-phase extraction	Protein precipitation
Sample aliquot volume	100 µL	100 µL	50 µL
Calibration model	Linear regression	Linear regression	Linear regression
Weighting factor	$1/x^2$	$1/x^2$	$1/x^2$
Total number of samples analyzed	871	178	139
Total number of samples reassayed	315 Analytical (36.2% of total number of samples analyzed)<br>95 ISR (10.9% of total number of samples analyzed)	25 ISR (14.0% of total number of samples analyzed)	1 Analytical (0.719% of total number of samples analyzed)<br>24 ISR (17.3% of total number of samples analyzed)
Sample storage conditions	-60 to -80°C	-60 to -80°C	-60 to -80°C
ISR	Required/Pass	Required/Pass	Required/Pass
Samples analyzed within known stability	Yes	Yes	Yes

CV, coefficient of variation; ETV, entecavir; ISR, incurred sample reanalysis; LLOQ, lower limit of quantification; TFV, tenofovir; ULOQ, upper limit of quantification.

References

1. Wong SG, Ramsden D, Dallas S, et al. Considerations from the Innovation and Quality Induction Working Group in Response to Drug-Drug Interaction Guidance from Regulatory Agencies: Guidelines on Model Fitting and Recommendations on Time Course for In Vitro Cytochrome P450 Induction Studies Including Impact on Drug Interaction Risk Assessment. *Drug Metab Dispos.* 2021;49(1):94-110.
2. Vieira ML, Kirby B, Ragueneau-Majlessi I, et al. Evaluation of various static in vitro-in vivo extrapolation models for risk assessment of the CYP3A inhibition potential of an investigational drug. *Clin Pharmacol Ther.* 2014;95(2):189-198.
3. Kenny JR, Ramsden D, Buckley DB, et al. Considerations from the Innovation and Quality Induction Working Group in Response to Drug-Drug Interaction Guidances from Regulatory Agencies: Focus on CYP3A4 mRNA In Vitro Response Thresholds, Variability, and Clinical Relevance. *Drug Metab Dispos.* 2018;46(9):1285-1303.
4. Agarwal S, Arya V, Zhang L. Review of P-gp inhibition data in recently approved new drug applications: utility of the proposed [I(1)]/IC(50) and [I(2)]/IC(50) criteria in the P-gp decision tree. *J Clin Pharmacol.* 2013;53(2):228-233.
5. Ellens H, Deng S, Coleman J, et al. Application of receiver operating characteristic analysis to refine the prediction of potential digoxin drug interactions. *Drug Metab Dispos.* 2013;41(7):1367-1374.
6. Zhang L, Zhang YD, Strong JM, Reynolds KS, Huang SM. A regulatory viewpoint on transporter-based drug interactions. *Xenobiotica.* 2008;38(7-8):709-724.
